# Barriers to male partner accompaniment and participation in maternal and child health care in Thika and Kiambu Level Five Hospital, Kenya

**DOI:** 10.11604/pamj.2023.44.189.38293

**Published:** 2023-04-20

**Authors:** Joseph Mukobe Okwako, Grace Wambura Mbuthia, Karani Magutah

**Affiliations:** 1 Department of Midwifery, Jomo Kenyatta University of Agriculture and Technology, Juja, Kenya,; 2 Department of Community Health Nursing, Jomo Kenyatta University of Agriculture and Technology, Juja, Kenya,; 3 Department of Medical Physiology, Moi University, Eldoret, Kenya

**Keywords:** Barriers, determinants, male involvement, male accompaniment, male participation, maternal and child health

## Abstract

**Introduction:**

the 1994 International Conference on Population and Development (ICPD) recommended that men should share responsibility and be actively involved in responsible parenthood, sexual and reproductive health. The level of male involvement in Kenya remains low despite growing evidence showing its benefits in maternal and newborn health. This study sought to determine factors influencing male partner involvement in maternal and child health with focus on accompaniment to maternal and child health (MCH) department.

**Methods:**

a qualitative study utilizing exploratory design was used to gather the views of men and nurse-midwives working in the MCH department of Thika and Kiambu County Teaching and Referral hospitals in January 2022. Qualitative data were collected from focused group discussions from nurses and men respectively. The number of participants per Focused Group Discussion (FGD) ranged between six to eight. The principal author moderated the FGD that were audio recorded and lasted between 60-90 minutes. Content analysis was used to analyse data following the five steps to yield themes using MAXQDA 2022 software.

**Results:**

five categories emerged as factors influencing male accompanying their spouses to MCH clinic: traditional gender norms, roles and beliefs, unfavorable MCH environment, work commitment, fear of HIV testing and men’s work commitment.

**Conclusion:**

traditional gender roles and norms, work commitment by men and unfavourable MCH set-up were key barriers identified that hinder men from accompanying their spouses to MCH clinic. There is need to develop an effective, feasible and sustainable intervention that will encourage male partners to accompany their spouses and participate in MCH services.

## Introduction

The role of men in access to care is believed to be critical given their role in decision-making. Men play a central role in improving maternal health [1]. For instance, male involvement can lead to increased access to health services for women, children, and men themselves, increased male partner support for family planning and reproductive health services, and increased uptake of Sexually Transmitted Infections (STI) and HIV services [2,3]. Interventions to engage men in maternal and newborn health can increase healthcare-seeking behavior and support more equitable couple communication and decision-making for maternal and newborn health [4]. Men as heads of the family control resources, consult soothsayers to determine the health seeking or treatment for pregnant women and serve as the final authority on where and when pregnant women should seek medical care [5]. Beyond that, they have no expectation of any further role during antenatal care and therefore find it unnecessary to attend clinics with their partners. It is believed that when men accompany their partners to health facilities, it helps them understand their maternal health problems and needs and eventually results in a greater understanding of their families and the community in general [6]. The Kenya Reproductive, Maternal, Newborn, Child and Adolescent Health (RMNCAH) Investment Framework advocates for the need to involve men to enhance the utilization of health care services by women [7].

In spite of these enormous available evidence, studies have reported low male attendance and involvement in maternal and child health departments in Kenya [8-10]. A Kenyan National Survey and an Eastern Kenyan study revealed that about 30% and 23% of men respectively accompany their spouses and or wives to the MCH clinic [8,9]. Several barriers have been cited that impede men from accompanying their spouses to the MCH department. They include negative cultural beliefs that maternal health is a female role while men are family providers; poor attitudes of healthcare providers, lack of space to accommodate male partners in health facilities; and the high cost associated with accompanying women to seek maternity care [9-13], lack of services targeting men, non-invitation to the clinic, long time spent at the clinic and lack of privacy at the clinics [10]. However, most of these studies utilized interviews and structured questionnaires to collect data, and men´s perspective was rarely captured. Moreover, only one study from the literature review which sought the perspectives of both HCWs and men was identified. This study, therefore, sought to explore factors influencing men from accompanying their spouses to MCH clinics from nurses and men in a different locality. Having a clear background understanding of the issues can better inform an appropriate intervention to promote male involvement in MCH services.

## Methods

**Study design:** this is a qualitative study that adopted an exploratory approach to determine factors hindering men from accompanying their partners to the MCH department.

**Study setting:** the study was conducted in Thika and Kiambu Level 5 Hospitals which are located in Kiambu County bordering Nairobi, Kenya. The two facilities are County teaching and referral hospitals offering curative, preventive, rehabilitative, and preventive health services. The study sites were purposively selected because of observed low levels of men accompanying their spouses to the MCH clinic (about three female accompaniments for sixty ANC attendance in a day).

**Study population and participant recruitment:** the study was conducted among nurse-midwives selected purposively from both Kiambu and Thika Level 5 Hospitals in Kiambu County which offer teaching and referral services. Male spouses of women attending MCH care services were also invited to participate. Purposive sampling was selected because participants shared common characteristics under study. In this case, the nurse-midwives had shared expert experience in the study topic while the men had their spouses attending ANC. Only nurses working in the MCH clinic were eligible for participation and were invited through WhatsApp, email, and phone calls. The nurse-in-charges facilitated the process by providing contacts of all the nurses. Participants in virtual Focused Group Discussions (FGDs) were requested to register using their official names while joining the Zoom link. Money used to purchase bundles was reimbursed at the end of the discussion as promised.

the consent process was established by providing information about the study and participants voluntarily requested to confirm their availability a few days prior to data collection. Nurses who volunteered to participate in the study were placed in three groups of 6-7 members. Participants in two FGDs were invited to attend virtual discussions while one FGD was held face-to-face in the MCH department. Women attending the MCH clinic were selected using homogeneous purposive sampling. Women who were at the waiting bay, staying with their spouses, were young, and having their first pregnancy were issued letters inviting their spouses to attend FGDs in the clinic. A sample size of 16 men was invited which was deemed sufficient to form two male FGDs comprising at least six participants as their views were to complement the perspectives of nurses whose views had already reached saturation in the third FGD. Six and seven men turned up for the FGDs on days one and two respectively [14].

**Data collection, management, and analysis:** data collection was done using focused group discussions composed of between 6-7 participants. The discussions were held in English. Data collection took place in December 2021 for nurse-midwives and in January 2022 for male participants. The principal researcher moderated three focused group discussions among nurse-midwives and two FGDs among men which were audio recorded and lasted 60-90 minutes. It is generally accepted that between six and eight participants are sufficient for an FGD while three focused group discussions are enough to identify all of the most prevalent themes within the data set [14,15]. Two FGDs among nurse-midwives were done virtually while the rest (three) were conducted face-to-face in the MCH department. Data saturation was achieved in the three FGDs from nurses and the male FGDs were used to complement the data. Data from focus group discussions were imported into MAXQDA version 2022 for content analysis following the five steps outlined to yield themes [16]. Both inductive and deductive coding was used. Initial codes were generated and new codes were generated after reading data. Codes were assigned to data. General refining of codes continued as analysis continued to subsequent FGDs and emerging sub-categories and categories generated. Finally, an analysis of the generated categories was done and a conclusion was drawn. Data validity was ensured through member checking of the transcribed data and generated themes. Furthermore, the principal researcher shared the audio-recorded FGDs, and transcripts and analyzed data with co-researchers, and their inputs were incorporated into the final analysis.

**Ethical considerations:** research ethical approval and permits were sought from Jomo Kenyatta University of Agriculture and Technology Research Ethics Committee (Ref. no. JKU/2/4/896B) and National Commission for Science, Technology, and Innovation (NACOSTI); Ref. no. 207751 respectively. Participants were informed about the nature and benefits of the study before obtaining written consent. Confidentiality was ensured by assigning personal identification numbers to study participants.

## Results

**Socio-demographic characteristics:** nearly all the nurses working in the MCH department who participated in the study were female (n=18). Most participants (n=13) had attained a diploma in Kenya Registered Community Health Nursing as the highest level of education. The findings also show that the majority of male respondents were casual workers of mostly low socio-economic status. Table 1 shows the socio-demographics of the participants.

**Table 1:** socio-demographic characteristics

Nurse-midwives (n=19)			Men respondents (n=13
**Gender**	Male	1	13
	Female	18	0
**Age**	20-30 years	4	3
	31-40 years	5	8
	> 40 years	10	2
**Marital status**	Single	5	0
	Married	12	11
	Divorced/separated	2	2
**Education level**	Primary	0	3
	Secondary	0	8
	College	15	2
	University	4	0
**Employment status**	Casual workers	0	9
	Employed	19	2
	Self-employed	0	2
**Working experience**	1-3 years	3	3
	4-6 years	11	8
	7-10 years	3	2
	> 10 years	2	0

**Barriers to low male accompaniment and participation in maternal and child health services:** the study sought to explore factors that lead to low female accompaniment by their spouses in antenatal and postnatal clinics. Six themes emerged to describe the views of both nurses and men as shown in Table 2. These were: traditional gender norms, roles, and beliefs, unfavorable MCH set-up, work commitment, fear of HIV testing, and lack of awareness.

**Table 2:** themes on barriers to male accompanying spouses to maternal and child health clinic

Theme	Codes
Traditional gender norms, roles and beliefs	Look shameful, responsibility of women to go to maternal and child health (MCH) clinic, ridiculed by fellow men
Work commitment	Lack of time, men-s nature of work, lack of permission from work, working far away from home
Unfavourable MCH set-up	Unsupportive staff; staff attitude, long ques and waiting time, staff gender in the MCH, lack of privacy, lack of services targeting men, Boredom at waiting bay
Fear of HIV testing	Tested for HIV
Lack of awareness	Lack of community awareness, lack of knowledge, lack of role models

**Traditional gender norms, roles, and beliefs:** the nurses revealed that it is a cultural belief, norm, and practice that pregnancy, childbirth, and care are the responsibility of women. This was reiterated by the majority of male respondents as indicated below. *It is a traditional belief that pregnancy and childbirth are supposed to be a woman´s affair. Since time immemorial, men have kept off the issue entirely because it is not a man´s affair. A woman is supposed to be helped by female friends and TBAs*,´ (participant 1, FGD 3-nurse). ‘*It is a societal tradition for men not to accompany their wives. As Africans, it is the responsibility of the woman to go to the clinic while pregnant and also take children for immunization*,´ (participant 5, FGD 2-man). ‘*In Africa, it is shameful to walk with a woman while pregnant. Men think that society will backbite them that they are walking with their wives to the clinic*,´ (participant 5, FGD 2-man).

**Work commitment:** work commitment was reported by most respondents as an impediment to accompanying their spouses to the MCH department. To the majority of men who worked in the informal sector, they pointed out a lack of time as they went to work in order to earn a living for their families. Furthermore, it was noted that the MCH department was operational only on weekdays for which it was difficult for men to get a day off. ‘*The clinic is not on Saturday or Sunday. If it was Saturday or Sunday, I may go say at 2.00 pm after working. In most cases, the clinic runs from 8.00 am to 2.00 pm on weekdays*,´ (participant 1, FGD 2-man). ‘*The working environment affects going to clinic. I can´t fail to go on duty because I am escorting my wife to clinic. Even if you tell the supervisor you are taking your wife to clinic, he/she will tell you to let the wife go alone while you report to work. The boss will not see the need to accompany your wife to clinic yet the wife is supposedly free*,´ (participant 1, FGD 1-man). *Another thing is you find most women in town are housewives and their husband are breadwinners who are largely self-employed. They leave their homes to go earn a living for their families thus lack time to accompany their partners to clinic*,´ (participant 2, FGD 1-man). ‘*There is also a case where the husband is working far away from the wife and therefore the woman has no choice but to go alone*,´ (participant 3, FGD 1-nurse). ‘*Maybe the man is a casual worker who is paid on daily basis, so if you tell him to accompany the wife to clinic, he imagines he will lose the wages so he will rather not go because if he does, the whole day will be wasted*,´ (participant 3, FGD 3-nurse).

**Fear of HIV testing:** a few respondents pointed out that some men do fear couple HIV testing which is a voluntary routine test among pregnant and lactating women. As a result, some men kept away from accompanying their spouses to the MCH department. ‘*I think some men fear HIV testing. You know the first visit you are tested for HIV. Even if you come with evidence of previous testing, they will still repeat testing. So, some men keep away from the first visit and wait for the results of their wives. If it turns out negative, they may go subsequent clinic visits*,´ (participant 2, FGD 2-man). *When it comes to ANC, some men may fear going to the clinic because of HIV testing because the woman has to be tested for HIV*,´ (participant 6, FGD 2-nurse).

**Unfavourable maternal and child health set-up:** respondents cited unsupportive staff; long queues and waiting time, absence of male staff in the MCH, and lack of male services as barriers to men accompanying their spouses to the MCH department.

***Unsupportive staff:*** *there are men who are willing to take their spouses to the clinic but when they go to ANC, healthcare workers will tell the man to remain outside. So, next time he will not accompany the wife to the clinic*,´ (participant 6, FGD 2-nurse). *Sometimes, they complain that even the attitude of health care workers is negative. They are not friendly to encourage men to attend the clinic frequently so they tend to relax and not accompany their partners. Even when they accompany their partners, a times they are not given a chance to enter the room together*,´ (participant 5, FGD 1-nurse).

***Long queues:*** *men don´t like lining up together with women and maybe the lines are so long and they want to go somewhere to look for money or work so they prefer somewhere where they can be seen immediately so that they can go look for money*,´ (participant 3, FGD 2-nurse). ‘*Men may also not want to attend clinic because of delay in receiving care. They don´t want to line-up*,´ (participant 6, FGD 3-nurse).

***Staff gender in the maternal and child health:*** *yes, absence of male staff can also contribute. It is really hard to see a male nurse working in MCH*,´ (participant 4, FGD 3-nurse). ‘*Some men are unable to disclose to female nurses. You will hear them say they don´t want to be attended to by female nurses. In most MCH clinics, we have female nurses working there, so men will not go to clinic at all because they know they will find female nurses*,´ (participant 2, FGD 2-nurse). ‘*If you went to the clinic and found the one attending to you is a female staff younger than you and you want to say something about the wife, you will be shy to talk. For example, issues of sexual intercourse*,´ (participant 7, FGD 2-man).

***Lack of services targeting men:*** ‘*talk of reproductive health needs, men just feel MCH is mother and child health*,´ (participant 5, FGD 3-nurse).

***Boredom at the waiting bay:*** *if you look at MCH in private and government hospitals, there is a lot of difference. I mean even while sited there you are occupied with a magazine, TV and you won´t miss news but in a public facility, there are just 1,2, or 3 posters from January to December so the men will come, read all those posters and get bored*,´ (participant 3, FGD 1-nurse).

**Lack of awareness:** *another reason is due to lack of awareness. The government has ignored men who have no knowledge on the need to accompany their partners*,´ (participant 6, FGD1-nurse). *Men lack information so they basically don´t know why they need to go to MCH clinic. Not being empowered enough is the reason why they don´t support their partners*,´ (participant 1, FGD 2-man).

## Discussion

In this study, only female nurses participated in the study which reflects the gender deployment disparity in most MCH departments. Moreover, casual and informal employment status among male participants reflects the low socio-economic status of clients who seek services in public hospitals that may impede male accompaniment. The findings of this study mirror in many aspects those of previous studies on factors influencing male involvement in maternal, newborn and child health services. In this study, traditional gender norms, roles and beliefs were identified as barriers to males accompanying their partners to the MCH department. Respondents indicated that maternal and child health services were a preserve for women while men went to work to earn a living. Male participants indicated that it was embarrassing to be seen with a pregnant woman in the clinic. Moreover, it was noted that the name maternal and child health left out the male component thus putting off men from going to the MCH department with their partners. This finding concurs with other previous studies which indicated that patriarchal community values and strong socio-cultural and gender norms hindered the adoption of male involvement [12,17-19]. In their study findings, it was noted that perceptions that pregnancy care is a female role while men are family providers hindered male involvement in MCH services [9].

Lack of time by men due to work commitment and an unsupportive MCH environment were other key barriers identified as hindering men from accompanying their spouses. Since most men respondents were casual labourers, they could not be given off from work to attend the MCH clinic. It was pointed out that the MCH clinic was operational during weekdays and preferably morning hours hence not accommodative to most men in casual employment. The characteristic long queue, boredom, unfriendly staff to male companions, and lack of health services addressing male needs discouraged men from attending the MCH department. In related study findings, poor attitudes of healthcare providers, lack of space to accommodate male partners in health facilities [9,12], the men´s nature of work, the lack of services targeting men, and long time spent at the clinic [12,13,18] were cited as barriers to male involvement in MCH services. Moreover, it was noted that while some men accompany their pregnant partners to the antenatal clinic and wait outside, very few men participate in antenatal consultations [20]. Other studies identified unfavourable opening hours of services and the high cost associated with accompanying women to seek maternity care [9]; low income and expenses incurred at ANC/PNC clinics, non-invitation to the clinic, and lack of privacy at the clinics [12]; lack of interest, men always busy [19], lack of separate waiting spaces for men, and being in a polygamous relationship [20,21]. As found in this study, fear of HIV testing [20,21], and lack of information among men on the importance of male involvement in sexual and reproductive health [13,22-24] were other barriers to male involvement in antenatal care.

**Study limitations:** some views presented in this study such as the setting of MCH do not reflect the perspective of private and faith-based health facilities. Virtual FGD´s limited observation of participants' body language. Furthermore, some participants in the virtual FGDs encountered technological disruptions which interfered with the timing and quality of the discussions. The findings are also limited to men of low-socioeconomic status since nearly all the male respondents were casual labourers.

## Conclusion

The findings of this study reflect the perspectives of men of low socio-economic status and healthcare workers working in public hospitals on barriers to low male accompaniment and participation in MCH services. These barriers include socio-cultural norms, nature of men’s work, unsupportive MCH environment and fear of HIV testing. Men accompanying their spouses to MCH department while being involved in the care of their partners can enhance the quality of maternal, child and reproductive health. There is need for stakeholders in health sector to design robust evidence-based interventions to overcome these barriers with a view to promoting male involvement in MCH services.

### What is known about this topic

- *A number of studies have identified barriers to male involvement in maternal, child, sexual and reproductive health though none was found to have been conducted in the study setting*.

### What this study adds

- *Unlike other previous similar studies, this study sought the perspectives of both the health care workers and men who had been invited to the hospital to determine barriers to male involvement thus providing a complementary, rich and validated data;*
- *This study also established that the naming of the department as MCH, limited working hours in the MCH department that excluded weekends and evenings are important barriers that hinder men from accompanying their spouses to the clinic in the study area*.
